# Supplementary material for: TopHat-Recondition: a post-processor for TopHat unmapped reads
Source: BMC Bioinformatics. 2016 May 4;17:199. doi: 10.1186/s12859-016-1058-x (PMC4855331; doi:10.1186/s12859-016-1058-x)
Supplement: Additional file 1 — Usage information and walk-through example. (PDF 166 kb) [file 12859_2016_1058_MOESM1_ESM.pdf]

Supplementary Materials for TopHat-Recondition 1.0

Christian Brueffer and Lao H Saal

Usage

TopHat-Recondition can be obtained from GitHub (https://github.com/cbrueffer/tophat-recondition/). Here we assume it is available as ~/tophat-recondition/tophat-recondition.py.
The only required argument for the software is a directory containing the TopHat output files accepted_hits.bam and unmapped.bam, such as the default TopHat tophat_out output directory. A full list of options can be obtained by running tophat-recondition.py without arguments.

```
$ ~/tophat-recondition/tophat-recondition.py
Usage:

tophat-recondition.py [-hqv] [-l logfile] tophat_output_dir [result_dir]

-h                  print this usage text and exit (optional)
-l                  log file (optional, default: result_dir/tophat-recondition.log)
-q                  quiet mode, no console output (optional)
-v                  print the script name and version, and exit (optional)
tophat_output_dir: directory containing accepted_hits.bam and unmapped.bam
result_dir:        directory to write unmapped_fixup.bam to (optional, default: tophat_output_dir)
```

By default, TopHat-Recondition will write the corrected unmapped read file unmapped_fixup.bam to the directory containing the input BAM files.

Example Run

To show the usage and operation of TopHat-Recondition, we use the workflow and data outlined in the TopHat tutorial:
Tutorial: http://ccb.jhu.edu/software/tophat/tutorial.shtml
Data: http://ccb.jhu.edu/software/tophat/downloads/test_data.tar.gz

Running TopHat

We extract the data and run TopHat 2.1.0 as instructed in the tutorial.

```
$ tar zxvf test_data.tar.gz
$ cd test_data
$ tophat -r 20 test_ref reads_1.fq reads_2.fq

[2015-10-30 12:58:40] Beginning TopHat run (v2.1.0)
-----------------------------------------------
[2015-10-30 12:58:40] Checking for Bowtie
      Bowtie version:  2.2.5.0
[2015-10-30 12:58:40] Checking for Bowtie index files (genome)..
  Found both Bowtie1 and Bowtie2 indexes.
[2015-10-30 12:58:40] Checking for reference FASTA file
[2015-10-30 12:58:40] Generating SAM header for test_ref
[2015-10-30 12:58:40] Preparing reads
   left reads: min. length=75, max. length=75, 100 kept reads (0 discarded)
  right reads: min. length=75, max. length=75, 100 kept reads (0 discarded)
[2015-10-30 12:58:40] Mapping left_kept_reads to genome test_ref with Bowtie2
[2015-10-30 12:58:41] Mapping left_kept_reads_seg1 to genome test_ref with Bowtie2 (1/3)
[2015-10-30 12:58:41] Mapping left_kept_reads_seg2 to genome test_ref with Bowtie2 (2/3)
[2015-10-30 12:58:41] Mapping left_kept_reads_seg3 to genome test_ref with Bowtie2 (3/3)
[2015-10-30 12:58:41] Mapping right_kept_reads to genome test_ref with Bowtie2
[2015-10-30 12:58:41] Mapping right_kept_reads_seg1 to genome test_ref with Bowtie2 (1/3)
[2015-10-30 12:58:41] Mapping right_kept_reads_seg2 to genome test_ref with Bowtie2 (2/3)
```

```
[2015-10-30 12:58:41] Mapping right_kept_reads_seg3 to genome test_ref with Bowtie2 (3/3)
[2015-10-30 12:58:41] Searching for junctions via segment mapping
[2015-10-30 12:58:41] Retrieving sequences for splices
[2015-10-30 12:58:42] Indexing splices
Building a SMALL index
[2015-10-30 12:58:42] Mapping left_kept_reads_seg1 to genome segment_juncs with Bowtie2 (1/3)
[2015-10-30 12:58:42] Mapping left_kept_reads_seg2 to genome segment_juncs with Bowtie2 (2/3)
[2015-10-30 12:58:42] Mapping left_kept_reads_seg3 to genome segment_juncs with Bowtie2 (3/3)
[2015-10-30 12:58:42] Joining segment hits
[2015-10-30 12:58:42] Mapping right_kept_reads_seg1 to genome segment_juncs with Bowtie2 (1/3)
[2015-10-30 12:58:43] Mapping right_kept_reads_seg2 to genome segment_juncs with Bowtie2 (2/3)
[2015-10-30 12:58:43] Mapping right_kept_reads_seg3 to genome segment_juncs with Bowtie2 (3/3)
[2015-10-30 12:58:43] Joining segment hits
[2015-10-30 12:58:43] Reporting output tracks
-----------------------------------------------
[2015-10-30 12:58:43] A summary of the alignment counts can be found in ./tophat_out/align_summary.txt
[2015-10-30 12:58:43] Run complete: 00:00:02 elapsed
```

Running TopHat-Recondition

TopHat writes its output files — `accepted_hits.bam` and `unmapped.bam` — to the directory `tophat_out`. We run TopHat-Recondition with this directory as argument. By not specifying a separate output directory, the corrected unmapped read file — `unmapped_fixup.bam` — will be written to the input directory `tophat_out`.

```
$ tophat-recondition.py tophat_out
2015-10-30 12:59:45 - Starting run of tophat-recondition 1.0
2015-10-30 12:59:45 - Command: tophat-recondition.py tophat_out
2015-10-30 12:59:45 - Current working directory: /home/chris/test_data
2015-10-30 12:59:45 - Writing logfile: tophat_out/tophat-recondition.log
2015-10-30 12:59:45 - Opening unmapped BAM file: tophat_out/unmapped.bam
2015-10-30 12:59:45 - Loading unmapped BAM file into memory: tophat_out/unmapped.bam
2015-10-30 12:59:45 - Setting missing 0x8 flag for unmapped read-pair: test_mRNA_150_290_0
2015-10-30 12:59:45 - Setting missing 0x8 flag for unmapped read-pair: test_mRNA_96_238_3
2015-10-30 12:59:45 - Setting missing 0x8 flag for unmapped read-pair: test_mRNA_75_235_21
2015-10-30 12:59:45 - Setting missing 0x8 flag for unmapped read-pair: test_mRNA_48_207_39
2015-10-30 12:59:45 - Setting missing 0x8 flag for unmapped read-pair: test_mRNA_94_291_40
2015-10-30 12:59:45 - Setting missing 0x8 flag for unmapped read-pair: test_mRNA_33_189_4a
2015-10-30 12:59:45 - Setting missing 0x8 flag for unmapped read-pair: test_mRNA_172_294_4f
2015-10-30 12:59:45 - Setting missing 0x8 flag for unmapped read-pair: test_mRNA_4_191_5d
2015-10-30 12:59:45 - Opening mapped BAM file: tophat_out/accepted_hits.bam
2015-10-30 12:59:45 - Standardizing flags of unmapped read: test_mRNA_5_197_46
2015-10-30 12:59:45 - Standardizing flags of unmapped read: test_mRNA_11_190_1a
2015-10-30 12:59:45 - Standardizing flags of unmapped read: test_mRNA_21_208_24
2015-10-30 12:59:45 - Standardizing flags of unmapped read: test_mRNA_23_186_42
2015-10-30 12:59:45 - Standardizing flags of unmapped read: test_mRNA_28_188_11
2015-10-30 12:59:45 - Standardizing flags of unmapped read: test_mRNA_28_206_1f
2015-10-30 12:59:45 - Standardizing flags of unmapped read: test_mRNA_30_231_3c
2015-10-30 12:59:45 - Standardizing flags of unmapped read: test_mRNA_33_223_4e
2015-10-30 12:59:45 - Standardizing flags of unmapped read: test_mRNA_44_225_1e
2015-10-30 12:59:45 - Standardizing flags of unmapped read: test_mRNA_44_193_3f
2015-10-30 12:59:45 - Standardizing flags of unmapped read: test_mRNA_46_195_17
2015-10-30 12:59:45 - Standardizing flags of unmapped read: test_mRNA_51_194_49
2015-10-30 12:59:45 - Standardizing flags of unmapped read: test_mRNA_57_231_8
2015-10-30 12:59:45 - Standardizing flags of unmapped read: test_mRNA_58_234_7
2015-10-30 12:59:45 - Standardizing flags of unmapped read: test_mRNA_58_220_3d
2015-10-30 12:59:45 - Standardizing flags of unmapped read: test_mRNA_65_238_2e
2015-10-30 12:59:45 - Standardizing flags of unmapped read: test_mRNA_69_229_23
2015-10-30 12:59:45 - Standardizing flags of unmapped read: test_mRNA_81_228_3a
2015-10-30 12:59:45 - Standardizing flags of unmapped read: test_mRNA_82_255_2
2015-10-30 12:59:45 - Standardizing flags of unmapped read: test_mRNA_89_230_b
2015-10-30 12:59:45 - Standardizing flags of unmapped read: test_mRNA_89_245_15
2015-10-30 12:59:45 - Standardizing flags of unmapped read: test_mRNA_92_266_43
2015-10-30 12:59:45 - Standardizing flags of unmapped read: test_mRNA_92_250_44
2015-10-30 12:59:45 - Standardizing flags of unmapped read: test_mRNA_97_275_26
2015-10-30 12:59:45 - Standardizing flags of unmapped read: test_mRNA_114_277_5b
2015-10-30 12:59:45 - Standardizing flags of unmapped read: test_mRNA_16_194_10
2015-10-30 12:59:45 - Standardizing flags of unmapped read: test_mRNA_131_260_33
2015-10-30 12:59:45 - Standardizing flags of unmapped read: test_mRNA_39_219_5c
2015-10-30 12:59:45 - Standardizing flags of unmapped read: test_mRNA_50_224_2d
2015-10-30 12:59:45 - Standardizing flags of unmapped read: test_mRNA_51_248_14
```

```
2015-10-30 12:59:45 - Standardizing flags of unmapped read: test_mRNA_128_252_36
2015-10-30 12:59:45 - Standardizing flags of unmapped read: test_mRNA_52_261_1b
2015-10-30 12:59:45 - Standardizing flags of unmapped read: test_mRNA_110_267_22
2015-10-30 12:59:45 - Standardizing flags of unmapped read: test_mRNA_111_268_d
2015-10-30 12:59:45 - Standardizing flags of unmapped read: test_mRNA_104_274_1c
2015-10-30 12:59:45 - Standardizing flags of unmapped read: test_mRNA_85_275_38
2015-10-30 12:59:45 - Standardizing flags of unmapped read: test_mRNA_75_277_3b
2015-10-30 12:59:45 - Standardizing flags of unmapped read: test_mRNA_125_280_48
2015-10-30 12:59:45 - Standardizing flags of unmapped read: test_mRNA_151_286_e
2015-10-30 12:59:45 - Standardizing flags of unmapped read: test_mRNA_125_293_60
2015-10-30 12:59:45 - Standardizing flags of unmapped read: test_mRNA_111_297_61
2015-10-30 12:59:45 - Standardizing flags of unmapped read: test_mRNA_145_300_37
2015-10-30 12:59:45 - Writing corrected BAM file: tophat_out/unmapped_fixup.bam
2015-10-30 12:59:45 - Program finished successfully.
```

Verifying the Result

The log details the modifications performed on the reads. To verify them, we can compare the original `unmapped.bam` file and the corrected `unmapped_fixup.bam` file.
In the original `unmapped.bam` file, unmapped read pairs cannot be identified by the bits set in their `FLAGS` fields (both reads having the "mate is unmapped" bit set), even though it clearly contains eight such pairs.

```
$ cd tophat_out
$ samtools view -f 0x8 unmapped.bam
$
$ samtools view unmapped.bam | cut -f 1 | sort | uniq --repeated
test_mRNA_150_290_0
test_mRNA_172_294_4f
test_mRNA_33_189_4a
test_mRNA_4_191_5d
test_mRNA_48_207_39
test_mRNA_75_235_21
test_mRNA_94_291_40
test_mRNA_96_238_3
```

The corrected `unmapped_fixup.bam` file shows the unmapped read pairs correctly.

```
$ samtools view -f 0x8 unmapped_fixup.bam
test_mRNA_150_290_0 77  * 0 0 * * 0 0
    TCCTAAAAAGTCCGCCTCGGTCTCAGTCTCAAGTAGAAAAAGTCCCGTTGGCGATCCGTCTACGTCCGAGTAAGA
    IIIIIIIIIIIIIIIIIIIIIIIIIIIIIIIIIIIIIIIIIIIIIIIIIIIIIIIIIIIIIIIIIIIIIIIIIII
test_mRNA_150_290_0 141 * 0 0 * * 0 0
    TACGTATTTGTCGCGCGGCCCTACGGCTGAGCGTCGAGCTTGCGATCCGCCACTATTACTTTATTATCTTACTCG
    IIIIIIIIIIIIIIIIIIIIIIIIIIIIIIIIIIIIIIIIIIIIIIIIIIIIIIIIIIIIIIIIIIIIIIIIIII
test_mRNA_96_238_3  141 * 0 0 * * 0 0
    GATGCAGCGACTGGACTATTTAGGACGATCGGACGGAGGAGGGCAGTAGGACGCTACGTATTTGGCGCGCGGACC
    IIIIIIIIIIIIIIIIIIIIIIIIIIIIIIIIIIIIIIIIIIIIIIIIIIIIIIIIIIIIIIIIIIIIIIIIIII
test_mRNA_96_238_3  77  * 0 0 * * 0 0
    GATCCGTCTACGTCCGCGTAAGATAATAAAGTACTAGTAGCGTATCGCAAGCTCGACGCTCAGCCGTAGGGCCGC
    IIIIIIIIIIIIIIIIIIIIIIIIIIIIIIIIIIIIIIIIIIIIIIIIIIIIIIIIIIIIIIIIIIIIIIIIIII
test_mRNA_75_235_21 77  * 0 0 * * 0 0
    ACGGACGGACTTAGAGCGTCAGATGCAGCGACTGGACTATTTAGCACGATCGGACTGAGGAGGGCAGTAGAACGT
    IIIIIIIIIIIIIIIIIIIIIIIIIIIIIIIIIIIIIIIIIIIIIIIIIIIIIIIIIIIIIIIIIIIIIIIIIII
test_mRNA_75_235_21 141 * 0 0 * * 0 0
    CCGTCTACGTCCGAGTAAGATAATAAAGTAATAGTGGCGTATCGCAAGCTCAACGCTCAGCCGTAGGGCCGTGCG
    IIIIIIIIIIIIIIIIIIIIIIIIIIIIIIIIIIIIIIIIIIIIIIIIIIIIIIIIIIIIIIIIIIIIIIIIIII
test_mRNA_48_207_39 77  * 0 0 * * 0 0
    GCCCCTACGGGGATGACGACTAGGACTACGGACGGATTTAGACCGTCAGATGCAGCGACTGGACTATTTAGGACG
    IIIIIIIIIIIIIIIIIIIIIIIIIIIIIIIIIIIIIIIIIIIIIIIIIIIIIIIIIIIIIIIIIIIIIIIIIII
test_mRNA_48_207_39 141 * 0 0 * * 0 0
    TAAGAGTGGCGTATCGCAAGATCGACGCTCAGCCGTAGGGCCGCGCGCCAAATACGTAGCGTCCTACTTCCCTCC
    IIIIIIIIIIIIIIIIIIIIIIIIIIIIIIIIIIIIIIIIIIIIIIIIIIIIIIIIIIIIIIIIIIIIIIIIIII
test_mRNA_94_291_40 141 * 0 0 * * 0 0
    GTCCCAAAAAGTCCGCCTCGATCCCAGTCTCAAGTAGAAAATGTCGCGTTGCCGATCCGTCTACGTCCCAGGAAG
    IIIIIIIIIIIIIIIIIIIIIIIIIIIIIIIIIIIIIIIIIIIIIIIIIIIIIIIIIIIIIIIIIIIIIIIIIII
test_mRNA_94_291_40 77  * 0 0 * * 0 0
    CAGATGCAGCGACTGTACTATTTAGGACGACCTGACTGAGGAGGGTAGTAGGACGCTACGTATTTGGCGCGCGGC
    IIIIIIIIIIIIIIIIIIIIIIIIIIIIIIIIIIIIIIIIIIIIIIIIIIIIIIIIIIIIIIIIIIIIIIIIIII
test_mRNA_33_189_4a 77  * 0 0 * * 0 0
    AGCCCGACGCTCAGCCGTAGGGCCGCGCGCCAAATAGGTAGCGTCCTACTGCCCTCCTCAGTCCGATCGTCCTAA
    IIIIIIIIIIIIIIIIIIIIIIIIIIIIIIIIIIIIIIIIIIIIIIIIIIIIIIIIIIIIIIIIIIIIIIIIIII
```

```
test_mRNA_172_294_4f   77  * 0 0 * * 0 0
    ACGGATGAGCGTCGAGCTTGCGATACGCCACTATTACTTTATTATCTTCCTCGGACGTAGACGGATCGCCAACGG
    IIIIIIIIIIIIIIIIIIIIIIIIIIIIIIIIIIIIIIIIIIIIIIIIIIIIIIIIIIIIIIIIIIIIIIIIIIIII
test_mRNA_33_189_4a 141 * 0 0 * * 0 0
    ACTGAGCTAGGACGTGCCACTACGGGGATTACCACTAGGGCTACGGACGGACTTAGAGCGTCAGATGCAGCGACT
    IIIIIIIIIIIIIIIIIIIIIIIIIIIIIIIIIIIIIIIIIIIIIIIIIIIIIIIIIIIIIIIIIIIIIIIIIIIII
test_mRNA_172_294_4f   141 * 0 0 * * 0 0
    CCCGTCCTAAAACGTCCGCCTCGATCCCAGTCTCAAGTAGAAAAAGTCCCGCTGCCGACCCGTCTACGTCCGAGT
    IIIIIIIIIIIIIIIIIIIIIIIIIIIIIIIIIIIIIIIIIIIIIIIIIIIIIIIIIIIIIIIIIIIIIIIIIIIII
test_mRNA_4_191_5d  77  * 0 0 * * 0 0
    CAAGCTCGACGCTCAGCCGTAGGGCCGCGCGCCAAATACGTAGTGTCCTACTGCCCTACTCAGTCCGATCGTCCT
    IIIIIIIIIIIIIIIIIIIIIIIIIIIIIIIIIIIIIIIIIIIIIIIIIIIIIIIIIIIIIIIIIIIIIIIIIIIII
test_mRNA_4_191_5d  141 * 0 0 * * 0 0
    ACTATCTGACGAGACTGGAGGCGCTTGCGACTGAGCTAGGACGTACCATTACGCGGATGACGACTAGGACTACGG
    IIIIIIIIIIIIIIIIIIIIIIIIIIIIIIIIIIIIIIIIIIIIIIIIIIIIIIIIIIIIIIIIIIIIIIIIIIIII
```

Example Use Case: Picard AddOrReplaceReadGroups

We can try to add a basic read group header to a merged file `merged.bam`, generated by merging the `accepted_hits.bam` with either the original `unmapped.bam` or the corrected `unmapped_fixup.bam` file.
With the original `unmapped.bam`:

```
$ samtools merge merged.bam accepted_hits.bam unmapped_fixup.bam
$ samtools sort merged.bam merged_refsort
$
$ java -jar ~/software/picard-tools-1.115/AddOrReplaceReadGroups.jar INPUT=merged_refsort.bam OUTPUT=
    merged_refsort_rg.bam RGLB=1 RGPL=illumina RGPU=NA RGSM=LU
[Thu Nov 12 10:40:56 CET 2015] picard.sam.AddOrReplaceReadGroups INPUT=merged_refsort.bam OUTPUT=
    merged_refsort_rg.bam RGLB=1 RGPL=illumina RGPU=NA RGSM=LU    RGID=1 VERBOSITY=INFO QUIET=false
    VALIDATION_STRINGENCY=STRICT COMPRESSION_LEVEL=5 MAX_RECORDS_IN_RAM=500000 CREATE_INDEX=false
    CREATE_MD5_FILE=false
[Thu Nov 12 10:40:56 CET 2015] Executing as chris@host on Linux 2.6.32-358.23.2.el6.x86_64 amd64;
    OpenJDK 64-Bit Server VM 1.7.0_45-mockbuild_2013_10_23_08_18-b00; Picard version: 1.115(30
    b1e546cc4dd80c918e151dbfe46b061e63f315_1402927010) JdkDeflater
INFO  2015-11-12 10:40:56 AddOrReplaceReadGroups  Created read group ID=1 PL=illumina LB=1 SM=LU

[Thu Nov 12 10:40:56 CET 2015] picard.sam.AddOrReplaceReadGroups done. Elapsed time: 0.00 minutes.
Runtime.totalMemory()=376963072
To get help, see http://picard.sourceforge.net/index.shtml#GettingHelp
Exception in thread "main" htsjdk.samtools.SAMFormatException: SAM validation error: ERROR: Record 143,
    Read name test_mRNA_150_290_0, Mapped mate should have mate reference name
  at htsjdk.samtools.SAMUtils.processValidationErrors(SAMUtils.java:452)
  at htsjdk.samtools.BAMFileReader$BAMFileIterator.advance(BAMFileReader.java:643)
  at htsjdk.samtools.BAMFileReader$BAMFileIterator.next(BAMFileReader.java:628)
  at htsjdk.samtools.BAMFileReader$BAMFileIterator.next(BAMFileReader.java:598)
  at htsjdk.samtools.SamReader$AssertingIterator.next(SamReader.java:514)
  at htsjdk.samtools.SamReader$AssertingIterator.next(SamReader.java:488)
  at picard.sam.AddOrReplaceReadGroups.doWork(AddOrReplaceReadGroups.java:107)
  at picard.cmdline.CommandLineProgram.instanceMain(CommandLineProgram.java:183)
  at picard.cmdline.CommandLineProgram.instanceMainWithExit(CommandLineProgram.java:124)
  at picard.sam.AddOrReplaceReadGroups.main(AddOrReplaceReadGroups.java:74)
```

As the error indicates, Picard AddOrReplaceReadGroups cannot process the merged BAM file containing the original `unmapped.bam` file. Running AddOrReplaceReadGroups with the `VALIDATION_STRINGENCY=LENIENT` option would work by simply ignoring the errors, but the result would be a BAM file with the same issues as the input files.
On the other hand, with the corrected `unmapped_fixup.bam` file, the command succeeds:

```
$ samtools merge merged.bam accepted_hits.bam unmapped_fixup.bam
$ samtools sort merged_fixup.bam merged_fixup_refsort
$
$ java -jar ~/software/picard-tools-1.115/AddOrReplaceReadGroups.jar INPUT=merged_fixup_sort.bam OUTPUT
    =merged_fixup_refsort_rg.bam RGLB=1 RGPL=illumina RGPU=NA RGSM=LU
[Wed Nov 11 17:43:33 CET 2015] picard.sam.AddOrReplaceReadGroups INPUT=merged_fixup_refsort.bam OUTPUT=
    merged_fixup_refsort_rg.bam RGLB=1 RGPL=illumina RGPU=NA RGSM=LU    RGID=1 VERBOSITY=INFO QUIET=
    false VALIDATION_STRINGENCY=STRICT COMPRESSION_LEVEL=5 MAX_RECORDS_IN_RAM=500000 CREATE_INDEX=false
     CREATE_MD5_FILE=false
[Wed Nov 11 17:43:33 CET 2015] Executing as chris@host on Linux 2.6.32-358.23.2.el6.x86_64 amd64;
    OpenJDK 64-Bit Server VM 1.7.0_45-mockbuild_2013_10_23_08_18-b00; Picard version: 1.115(30
    b1e546cc4dd80c918e151dbfe46b061e63f315_1402927010) JdkDeflater
```

```
INFO  2015-11-11 17:43:33 AddOrReplaceReadGroups  Created read group ID=1 PL=illumina LB=1 SM=LU

[Wed Nov 11 17:43:33 CET 2015] picard.sam.AddOrReplaceReadGroups done. Elapsed time: 0.00 minutes.
Runtime.totalMemory()=376963072
```

In conclusion, the `unmapped_fixup.bam` or `merged_fixup.bam` files containing the corrected unmapped reads can be used as input for further BAM processing and analysis software, e.g., Picard, GATK, or quality assessment software like RNA-SeQC (`https://www.broadinstitute.org/cancer/cga/rna-seqc`). This can be done without the need for reduced strictness requirements that could mask other problems in the data file, or discarding non-conforming reads from the file, both of which would lead to ignoring potentially useful data. The corrected files can also be deposited in a sequencing archive like NCBI Gene Expression Omnibus (GEO) or the European Nucleotide Archive (ENA), without the need for others to deal with the problems described in this paper.
